# In Search of Monocot Phosphodiesterases: Identification of a Calmodulin Stimulated Phosphodiesterase from *Brachypodium distachyon*

**DOI:** 10.3390/ijms22179654

**Published:** 2021-09-06

**Authors:** Mateusz Kwiatkowski, Aloysius Wong, Anna Kozakiewicz-Piekarz, Christoph Gehring, Krzysztof Jaworski

**Affiliations:** 1 Department of Plant Physiology and Biotechnology, Nicolaus Copernicus University, Lwowska St. 1, 87-100 Torun, Poland; jaworski@umk.pl; 2 Department of Biology, College of Science and Technology, Wenzhou-Kean University, 88 Daxue Road, Ouhai, Wenzhou 325060, China; alwong@kean.edu; 3 Zhejiang Bioinformatics International Science and Technology Cooperation Center, Wenzhou-Kean University, Wenzhou 325060, China; 4 Department of Biomedical and Polymer Chemistry, Faculty of Chemistry, Nicolaus Copernicus University, Gagarina St. 7, 87-100 Torun, Poland; akoza@umk.pl; 5 Department of Chemistry, Biology and Biotechnology, University of Perugia, Borgo XX giugno, 74, 06121 Perugia, Italy; christophandreas.gehring@unipg.it

**Keywords:** phosphodiesterase (PDE), cAMP, cGMP, calmodulin (CaM), calcium ions, protein–protein interactions, *Brachypodium distachyon*

## Abstract

In plants, rapid and reversible biological responses to environmental cues may require complex cellular reprograming. This is enabled by signaling molecules such as the cyclic nucleotide monophosphates (cNMPs) cAMP and cGMP, as well as Ca^2+^. While the roles and synthesis of cAMP and cGMP in plants are increasingly well-characterized, the “off signal” afforded by cNMP-degrading enzymes, the phosphodiesterases (PDEs), is, however, poorly understood, particularly so in monocots. Here, we identified a candidate PDE from the monocot *Brachypodium distachyon* (BDPDE1) and showed that it can hydrolyze cNMPs to 5′NMPs but with a preference for cAMP over cGMP in vitro. Notably, the PDE activity was significantly enhanced by Ca^2+^ only in the presence of calmodulin (CaM), which interacts with BDPDE1, most likely at a predicted CaM-binding site. Finally, based on our biochemical, mutagenesis and structural analyses, we constructed a comprehensive amino acid consensus sequence extracted from the catalytic centers of annotated and/or experimentally validated PDEs across species to enable a broad application of this search motif for the identification of similar active sites in eukaryotes and prokaryotes.

## 1. Introduction

Cyclic nucleotide monophosphates (cNMPs), such as 3′,5′-cyclic adenosine monophosphate (cAMP) and 3′,5′-cyclic guanosine monophosphate (cGMP), are well-established as essential signaling and effector molecules in both prokaryotes and eukaryotes [1,2]. The presence and physiological relevance of cyclic nucleotides in plants was controversial for a long time because of their low concentrations as compared to animals (for review, see Reference [3]). However, recent evidence has established cNMP-dependent processes in plants ranging from signaling to the control of transcription, translation and metabolism [4,5,6,7,8] The cyclic NMP levels are dependent on the activities of two key enzymes, the cyclic mononucleotide cyclases and cyclic mononucleotide phosphodiesterases (PDEs). Adenylyl (AC) and guanylyl (GC) cyclases catalyze the conversions of ATP and GTP to the respective products cAMP and cGMP. Cyclic AMP and cGMP, in turn, serve as “on signals” for cNMP-dependent cellular processes. Consequently, the “off signal”, the hydrolysis of cAMP or cGMP to AMP or GMP, is enabled by PDEs that convert cNMP into 5′NMP by hydrolyzing the 3′-phosphodiester bonds [9]. Although cNMPs and plant nucleotide cyclases are increasingly recognized as essential components of many plant functions [10,11,12,13,14,15], our understanding of plant PDEs is still scant. To date, only two proteins in dicotyledonous [16,17] and one in liverwort [18] have been reported to have PDE activity, and notably, no PDE has yet been found in monocotyledonous plants. Our knowledge of the physiological processes in which cNMP participate is still expanding, and the discovery and characterization of novel PDEs will yield insights into the complex functions of cNMP-dependent processes at the molecular and systems levels.

In plants, cAMP and cGMP signaling may be tuned by Ca^2+^, ROS (reactive oxygen species) or NO (nitric oxide) [1], and interactions between Ca^2+^ and cNMP have been reported to modulate physiological processes, e.g., via cyclic nucleotide-gated channels (CNGC) that can be considered intersections of cNMP and Ca^2+^ signaling [19]. Crosstalk between Ca^2+^ and cNMP can also occur via nucleotide cyclases, as seen in phytosulfokine (PSK) signaling, where the GC activity of the hormone receptor (PSKR1) is significantly enhanced by Ca^2+^ [20]. In the case of plant ACs, there is, as yet, no experimental evidence of interactions with Ca^2+^, but it is well-documented that animal ACs are directly and indirectly regulated by Ca^2+^ and/or calmodulin [21]. Considering the various regulatory regions of animal PDEs, especially in group 1 phosphodiesterases (PDE1), which are allosterically regulated by calmodulin [22], it is likely that Ca^2+^ may also modulate the plant PDE activity and, hence, contribute to cNMP homeostasis. In view of recent reports on plant PDEs, it may turn out that they act as intramolecular regulators moonlighting in complex multifunctional proteins [17,18].

In this study, we identified and characterized the activity of a candidate PDE (BDPDE1) from the monocot *Brachypodium distachyon* and then derived a comprehensive and inclusive search term (amino acid motif) based on the catalytic centers of annotated PDEs across species to enable the discovery of similar PDE centers that might be hidden in complex multidomain proteins, particularly in plants, where investigations on the mechanisms that govern cNMP metabolism are still in their infancy.

## 2. Results and Discussion

### 2.1. Identification and Characterization of PDE Activity in BDPDE1

Here, we set out to discover the as yet elusive monocot phosphodiesterases that are key to the regulation of the cAMP and cGMP levels in the cell. First, we explored if the *Arabidopsis thaliana* PDE ATCN-PDE1 (At1g17330) [16] had any orthologs in the monocot *B. distachyon*. When the sequence of ATCN-PDE1 was used to query the *B. distachyon* proteome, we found an ortholog, BDPDE1 (NCBI: XP_003574089.2), with 66% identical amino acids covering 69% of the protein. A comparative analysis of the BDPDE1 sequence with its ortholog showed that both proteins belong to the family of YpgQ-like proteins, which are members of the highly conserved HD superfamily (Pfam 01966). This group of enzymes exhibits broad substrate specificity, acting as a 2′-nucleotidase, pyrophosphohydrolase, phosphatase or 2′,3′-cyclic phosphodiesterase [23,24,25]. YpgQ-like proteins contain an HD motif, which is shared with the family of metal hydrolases and class I PDEs; therefore, proteins referred to as YpgQ hydrolases can function as putative phosphodiesterases. This is consistent with the fact that ATCN-PDE1, which participates in the opening of stomata in response to UVA, reduces the pool of cGMP [16].

**Figure 1:**
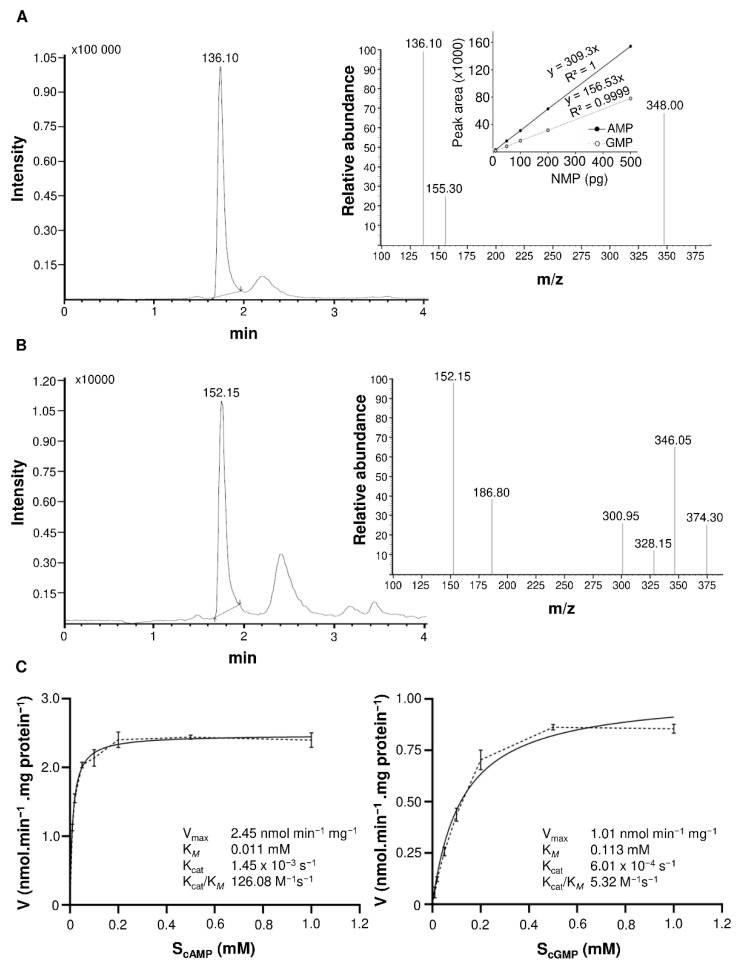
Kinetic parameters of BDPDE1. (**A**) Ion chromatogram of AMP with the inset showing the daughter AMP ion at *m/z* 136.10 [M + H]^+^. The fragmented product ion was used for quantitation of the phosphodiesterase activity. Inside is a calibration curve for AMP and GMP. (**B**) Ion chromatogram of GMP with the inset showing the daughter GMP ion at *m/z* 152.15 [M + H]^+^. The fragmented product ion was used for quantitation of the phosphodiesterase activity. (**C**) Michaelis–Menten plots for the cyclic nucleotide phosphodiesterase activity of BDPDE1. The V_max_ for a cAMP substrate was 2.45-nmol AMP min^−1^ mg protein^−1^ and a K*_M_* of 0.011 mM, respectively. The V_max_ for a cGMP substrate was 1.01-nmol GMP min^−1^ mg protein^−1^ and a K*_M_* of 0.113 mM, respectively. The reaction time was 25 min, and the standard reaction mixture contained: 3-mM Tris-HCl (pH 8.0), cAMP or cGMP in a concentration ranging from 0.01 mM to 1 mM, 0.1 % (*v/v*) 2-mercaptoethanol, 5 µg of GST-BDPDE1 and 0.5-mM MgCl_2_ and MnCl_2_ in a final volume of 100 μL.

The functional evaluation of the catalytic activity of the candidate PDE was done in vitro by enzymatic assays using cAMP and/or cGMP as a substrate. Reaction products AMP and GMP were detected and quantified using the sensitive liquid chromatography tandem mass spectrometry (LC-MS/MS) method (Figure 1A,B). BDPDE1 generates both AMP and GMP as a result of the reaction; however, BDPDE1 shows a higher affinity towards cAMP (Figure 1C). The V_max_ for cAMP as a substrate was 2.45-nmol AMP min^−1^ mg protein^−1^ and a K*_M_* of 0.0115 mM, whereas in the case of cGMP as a substrate, the V_max_ was 1.01-nmol GMP min^−1^ mg protein^−1^, and the affinity for cGMP was almost 10-fold lower (K*_M_* of 0.1133 mM). The activities obtained by BDPDE1 were somewhat lower compared with those of the *A. thaliana* ortholog (V_max_ 58.22-nmol min^−1^ mg protein^−1^ and K*_M_* 0.0258 mM) [16] and comparable to the *A. thaliana* K^+^ transporter ATKUP5 (V_max_ 1.17-nmol AMP min^−1^ mg protein^−1^ and K*_M_* 0.0053 mM) [17], which also contained a moonlighting PDE domain.

**Figure 2:**
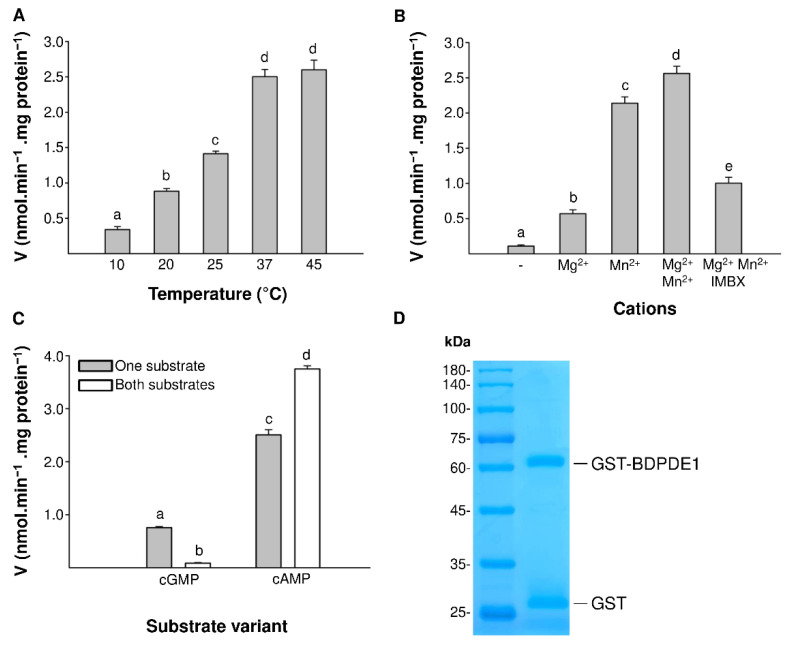
Characterization of the biochemical properties of BDPDE1. (**A**) Influence of temperature on the PDE activity. The enzymatic activity was tested in the temperature range from 10 °C to 45 °C. The reaction time was 25 min in the standard reaction mixture containing 0.1-mM cAMP and 5 µg of GST-BDPDE1. (**B**) Effect of divalent cations and inhibitor on the PDE activity of GST-BDPDE1. The reaction was carried out in the standard reaction mixture containing 0.1-mM cAMP, 5 µg of GST-BDPDE1 and, depending on the reaction variant, 0.5-mM MgCl_2_ and/or MnCl_2_, and 50-μM IMBX was added. (**C**) Substrate specificity of GST-BDPDE1. The reaction was performed in the presence of 5 µg of GST-BDPDE1, and depending on the substrate variant, 0.1-mM cAMP and/or 0.1-mM cGMP was added. In all the error bars, different letters indicate significant differences at *p* < 0.05. (**D**) The purified GST-BDPDE1 protein (4 μg) was analyzed by SDS-PAGE.

Next, we characterized the influence of temperature, ion cofactors and the PDE inhibitor IBMX (3-Isobutyl-1-methylxanthine) on the catalytic properties of the enzyme. The enzymatic activity was tested in the temperature range from 10 °C to 45 °C. As the temperature increased, a significant increase in activity was noticeable, tailing off at 37 °C (Figure 2A). These results are comparable with the experiments carried out on the cGMP-stimulated PDE from calf livers [26], where the highest catalytic efficiency was achieved at 37 °C. Subsequently, enzymatic reactions were carried out in the presence or absence of the cofactors 0.5-mM Mg^2+^ and 0.5-mM Mn^2+^. Divalent cations stimulated the enzymatic activity (Figure 2B), and the addition of both the Mg^2+^ and Mn^2+^ ions resulted in the PDE reaching the highest activity. In the case of animal PDEs, there are structural reasons for the dependence on two metal ions: the coordinated conserved histidine and aspartate residues that interact with Zn^2+^ and the weaker binding of Mg^2+^ in the catalytic pocket [27,28]. It appears that, in BDPDE1, the role of the metal ion is taken over by Mn^2+^, since, in its presence, the enzyme has >3-fold higher activity than in the presence of Mg^2+^. A similar effect was observed previously in MPCAPE-PDE (*Marchantia polymorpha*), where among the tested ions, the enzymatic activity was higher in the presence of Mn^2+^ [18]. Furthermore, the use of a nonselective PDE inhibitor IBMX at a concentration of 50-µM reduced the enzymatic activity two-fold.

The kinetics of BDPDE1 showed that the enzyme is capable of hydrolyzing both cAMP and cGMP. To check if there is a competition of cAMP and cGMP at the catalytic core of the PDE domain, assays measuring the BDPDE1 activity in the presence of two substrates simultaneously were performed, both in the same 0.1-mM concentrations. BDPDE1 favored the cAMP substrate, having a greater V_max_ and affinity for it. Interestingly, when both substrates are present in the reaction mixture, the activity of this enzyme towards cAMP increases >1.5-fold (Figure 2C). At the same time, the cGMP hydrolysis is negligible, decreasing nine-fold compared to the base value. The activity of cAMP hydrolysis was activated by the addition of cGMP, which is similar to a rat PDE2, where cGMP binds to an allosteric site regulating the enzymatic activity [29,30]. These results indicate that, while acting primarily as a plant cAMP PDE, BDPDE1 may also function as a cAMP-inhibited cGMP PDE. It is therefore conceivable that cAMP-mediated signal transduction can also cross-regulate the signaling strength of cGMP.

### 2.2. The Calmodulin/Ca^2+^ Complex Stimulates BDPDE1 Activity

**Figure 3:**
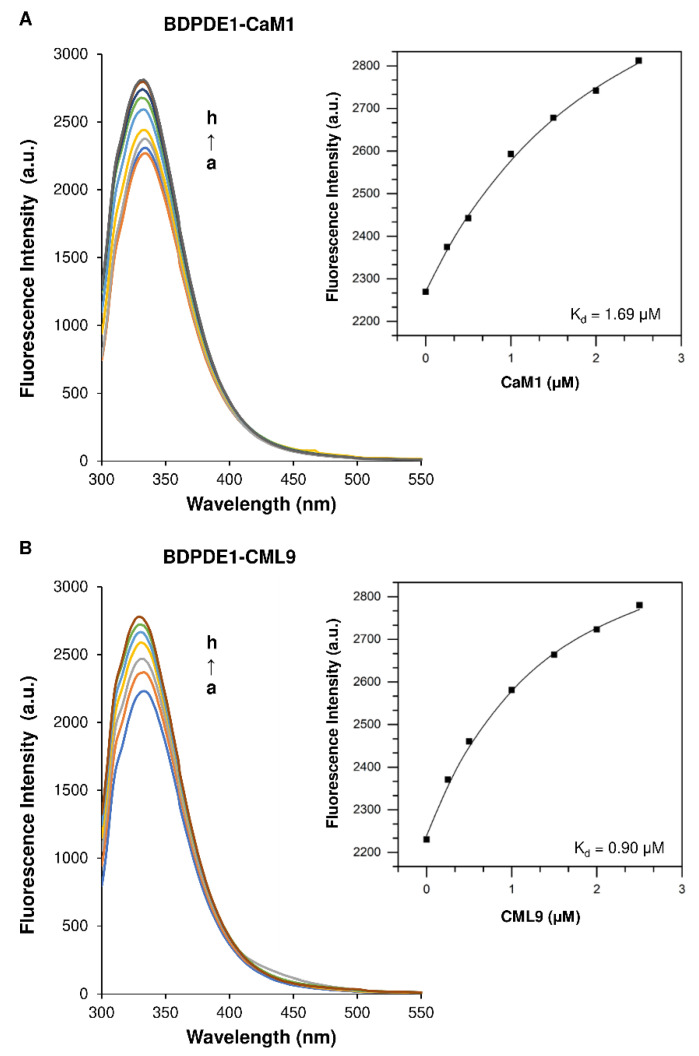
The fluorescence data of BDPDE1 in the presence of (**A**) CaM1 and (**B**) CML9 at 37 °C. (left). Emission spectra of BDPDE1 in the presence of CaM1 and CML9 (right) and the dependence of the fluorescence intensity on the CaM1 and CML9 concentrations. The concentration of BDPDE1 was 0.5-μM. The concentrations of CaM1 and CML9 from “a” to “h” were 0, 0.25, 0.5, 1.0, 1.5, 2.0 and 2.5 μM, respectively.

Calcium-dependent cellular processes are regulated through intracellular Ca^2+^-binding proteins, of which the best-studied are calmodulin (CaM), calmodulin-like proteins (CMLs) and calcium-dependent protein kinases (CDPKs). These proteins bind Ca^2+^ ions through the EF hand motif, a conserved helix–loop–helix structure that binds a single Ca^2+^ ion, thereby causing a change in the conformation that, in turn, can activate target proteins or cause self-activation [31,32,33,34]. Since it was reported that CaM/Ca^2+^ affects the activity of group I animal PDEs and the PDE domain of the ATKUP5 [17,35] and that a protein sequence analysis revealed that BDPDE1 also contains a predicted CaM-binding site between amino acids 50 and 70, we investigated the possible interactions of CaM and CaM-like isoforms with plant PDE using fluorescence spectroscopy. Due to the high structural similarities of CaM isoforms, we chose one representative of the CaM, using CaM1 and CaM-like isoform 9 (CML9). The BDPDE1 fluorescence spectra in the presence of CaM1 and CML9 at 37 °C are shown in Figure 3. The control emission spectra of BDPDE1, the buffer (TRIS), CaM1 and CML9 are shown in Supplementary Figure S1.

The results demonstrated that, at an excitation with 280 nm, BDPDE1 has a distinct peak of fluorescent emission at 333 nm deriving from tryptophan residues. CaM1 and CML9 lack tryptophan residues and instead contain tyrosine residues, which makes it possible to study their interaction with BDPDE1. We found that the plant PDE fluorescence intensity increases with the increasing concentration of CaM1 and CML9, while the maximum emission wavelength does not change. The results are indicative of an interaction between investigated CaM1 and CML9 with BDPDE1. The fluorescence data of the formation of the BDPDE1-CaM1 and BDPDE1-CML9 complexes were analyzed using the following equation assuming a 1:1 stoichiometry [36]:

(1) $$\frac{PL}{{\left[P\right]}_{t}}=\frac{{\left[P\right]}_{t}+{\left[L\right]}_{a}+K_{d}-\sqrt{{\left({\left[P\right]}_{t}+{\left[L\right]}_{a}+K_{d}\right)}^{2}-4{\left[P\right]}_{t}{\left[L\right]}_{a}}}{2{\left[P\right]}_{t}}$$

where *K_d_* is the dissociation constant, [*P*]*_t_* is the concentration of the protein, [*L*]*_a_* is the total concentration of the ligand and [*PL*] is the concentration of the protein–ligand complex. The obtained fluorescence data were fitted to the one-site binding model with an applied nonlinear least-squares regression using OriginPro software (Figure 3).

Our studies revealed that CaM1 and CML9 strongly bind to BDPDE1, as evidenced by the binding constant (*K_b_*) value in the order of 10^6^ M^−1^. We observed that CaM1 has a lower affinity (0.59 ± 0.09 L/mol), while CML9 binds to BDPDE1 in a higher binding constant of 1.11 ± 0.19 L/mol. We also determined that the dissociation constants (*K_d_*) value for CaM1 and CML9 were 1.69 ± 0.28 μM and 0.90 ± 0.16 μM, respectively.

**Figure 4:**
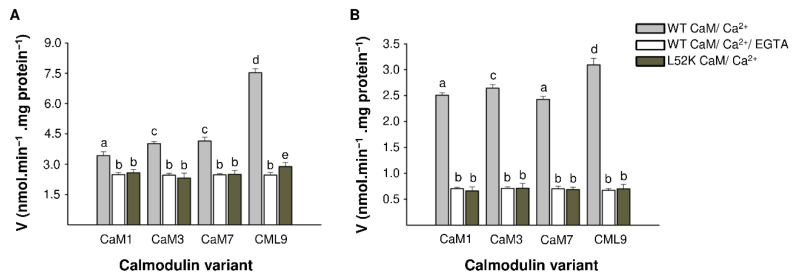
Regulation of the enzyme activity by CaM and CaM-like isoforms. (**A**) BDPDE1 and BDPDE1^L52K^ activity in the presence of various CaM isoforms, which were in active or inactive complexes (addition of 1-mM EGTA chelate). The reaction was carried out for 25 min in the standard reaction mixture containing 0.1-mM cAMP, 0.625-μM GST-BDPDE1, 2-μM of each CaM isoform, 12.5-μM CaCl_2_ and 1-mM EGTA, depending on the variant. Different letters indicate significantly different data between the groups at *p* < 0.05. (**B**) The BDPDE1 and BDPDE1^L52K^ activity in the presence of various CaM isoforms, where 0.1-mM cGMP was used as a substrate.

Further, we examined the influence of the four CaM isoforms (1, 3, 7 and CML9) on the PDE activity. Since calmodulins are highly conserved among plant species and show no significant differences in their structures (Supplementary Figure S2), CaM isoforms derived from *A. thaliana* were used in the experiment. All CaM isoforms stimulated both cAMP and cGMP hydrolysis after the formation of the active CaM/Ca^2+^ complex in the presence of 10-μM Ca^2+^ ions (Figure 4A,B). The highest increase in activity for both cAMP and cGMP hydrolysis were noted with the addition of CML9. For individual CaM isoforms, the increase in PDE activity was 3.5-fold for cGMP (Figure 4B) and 1.5-fold for cAMP (Figure 4A), and for CML9, it was 4.5-fold for cGMP and three-fold for cAMP. After the addition of 1-mM EGTA, which chelates Ca^2+^ ions, the active CaM/Ca^2+^ complex was not formed, and the presence of CaM isoforms did not affect the reaction.

We further investigated the effect of the CaM-binding on BDPDE1 activity with site-directed mutagenesis. In the determined CaM-binding site, leucine at position 52 was mutated to lysine, which disrupts CaM-binding [37]. This substitution resulted in no increase in BDPDE1 activity in the presence of an active CaM/Ca^2+^ complex. No CaM isoform had an effect on the cAMP and cGMP hydrolysis reactions; however, a slight increase in BDPDE1 activity was noted for CML9. This may be due to the fact that CMLs show significant structural differences from CaMs, and their binding may also alter the conformation of plant proteins in other ways.

**Figure 5:**
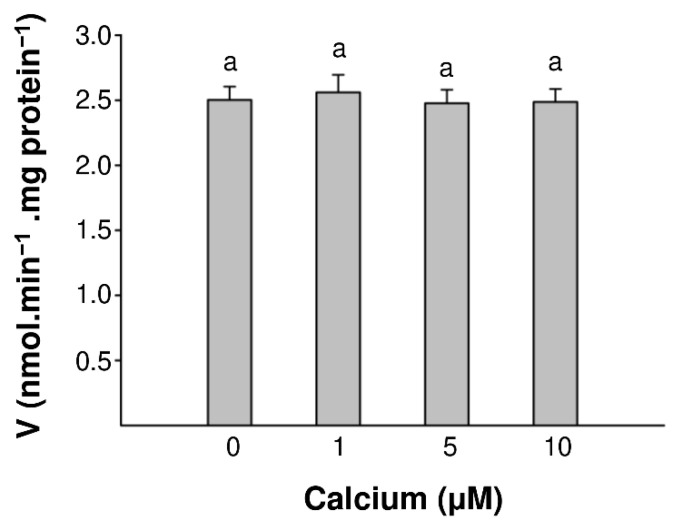
Impact of Ca^2+^ ions on the GST-BDPDE1 activity. AMP generated in vitro after 25 min by 5 µg of GST-BDPDE1 in the presence of 0.1-mM cAMP, 0.1 % (*v/v*) 2-mercaptoethanol, 0.5-mM MgCl_2_ and MnCl_2_ and CaCl_2_ in the concentration range from 0 to 12.5 µM. The same letters indicate no statistically significant differences at *p* < 0.05.

Given that, in plants, many proteins have been reported as calcium-binding (e.g., CDPKs (calcium-dependent protein kinases), SnRKs (SNF-related serine/threonine-protein kinases) or SOSs (salt overly sensitive) and responsive to calcium [32,38], we wanted to determine if we could rule out the possibility of a nonspecific increase in activity due to the presence of Ca^2+^. Therefore, we tested the effect of different concentrations of Ca^2+^ ions (0–10 µM) on the BDPDE1 enzymatic activity and noted no significant differences in the enzyme activities (Figure 5). This is consistent with no unspecific binding of Ca^2+^ to BDPDE1.

### 2.3. Probing the Catalytic Center of BDPDE1 and Construction of an Expanded PDE Search Motif

**Figure 6:**
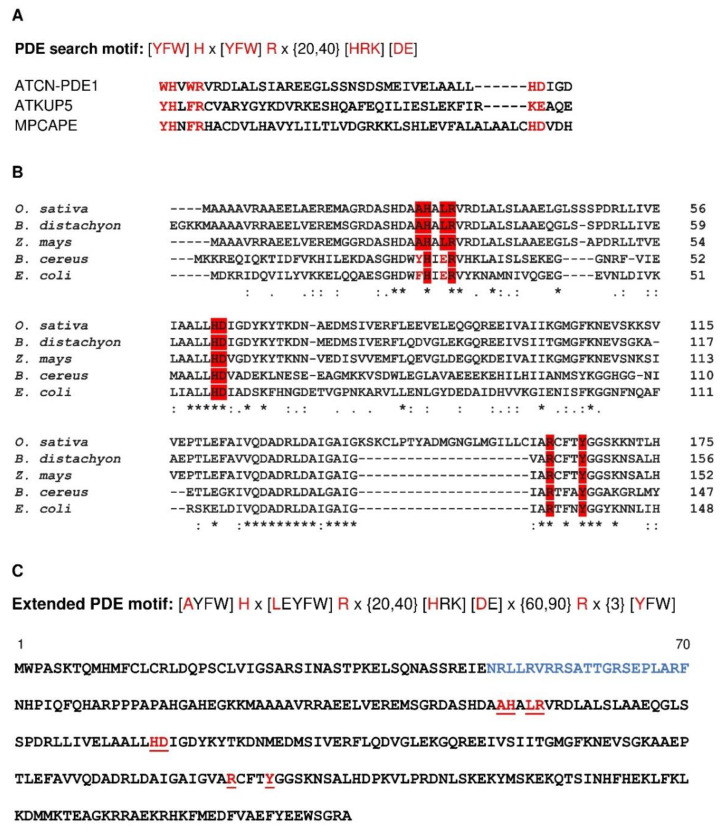
(**A**) Alignment of the known PDE centers of ATCN-PDE1 (At1g17330), ATKUP5 (At4g33530) and MPCAPE (Mapoly0068s0004). An * (asterisk) indicates positions that have a single, fully conserved residue. A: (colon) indicates conservation between groups of strongly similar properties. A. (period) indicates conservation between groups of weakly similar properties. (**B**) Alignment of the BDPDE1 orthologs in monocots *O. sativa* (NCBI: EEC67204.1) and *Z. mays* (NCBI: NP_001148226.1) and prokaryotes *B. cereus* (NCBI: CUB11224.1) and *E. coli* (NCBI: OJR83528.1). (**C**) Sequence of the extended PDE motif and amino acid sequence of the BDPDE1 protein (NCBI: XP_003574089.2). The key amino acids of the PDE motif are marked as red letters, and the calmodulin binding site is marked as blue letters.

Previous studies using a search motif approach to discover novel plant PDEs have shown that it might identify functional PDEs that moonlight in complex, multidomain proteins [17]. The motif [YFW]Hx[YFW]Rx{20,40}[HRK][DE] reported previously [17] is present in the catalytic centers of all the previously experimentally confirmed plant PDEs (Figure 6A). However, the PDE motif that was deliberately made stringent to identify candidate PDEs with high probability may be insufficiently broad, as no monocot orthologs harbor the full motif. Since BDPDE1 is functionally active in vitro, we probed the PDE catalytic center through mutagenesis studies, which are guided by a combination of sequence and structural analyses, to reveal other functionally important amino acids, especially in monocots. Through a sequence analysis, we found that, in BDPDE1, which is the monocot ortholog of ATCN-PDE1, alanine (A) and leucine (L) rather than an aromatic amino acid (YFW), occupy position 1 and 4 of the motif. Notably, these two amino acids are also conserved in other monocot orthologs, such as *Oryza sativa* (NCBI: EEC67204.1) and *Zea mays* (NCBI: NP_001148226.1) (Figure 6B). To account for a broader identification of PDE active sites in monocots and dicots, we therefore decided to include the conserved A and L amino acids in an expanded PDE motif.

We then probed the structure at the catalytic center of BDPDE1 to visualize how catalysis at the predicted PDE center might occur. Since the BDPDE1 crystal structure is unknown, we employed a homology modeling strategy to build a 3D model for BDPDE1 using the crystal structure of a metal-dependent HD domain-containing hydrolase from *Bacillus halodurans* (PDB ID: 3DTO) as the template. This has a 37.04% identity to BDPDE1 at the region between R80 to A315 and covers 91% of the queried amino acids. There is, however, no suitable template structure to model the N-terminal region of BDPDE1 that contains the predicted CaM-binding site at N50–F70 (Figure 6C) and was thus omitted from the structural analysis.

**Figure 7:**
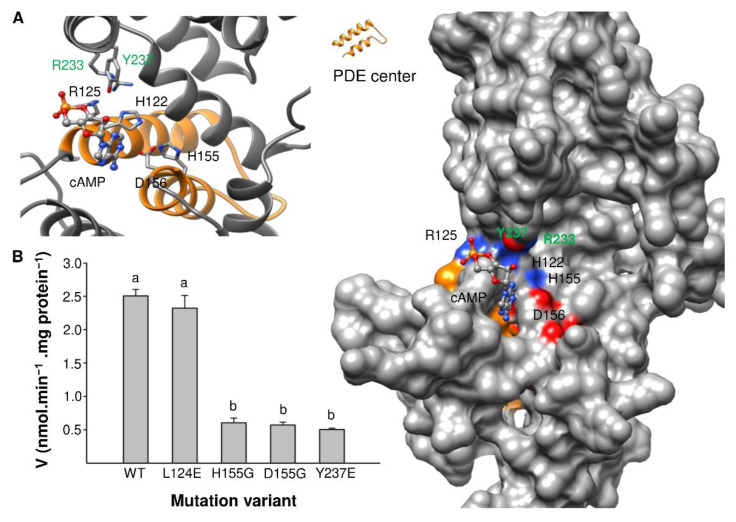
Computational assessment of the PDE catalytic center in BDPDE1. (**A**) The PDE center of BDPDE1 is shown as orange in the surface model (right) and as individual atoms in the ribbon model (left). The PDE center is solvent-exposed and occupies a distinct cavity that docks cAMP with a binding affinity of −4.9 kcal/mol. Key amino acids in the original PDE motif [YFW]**H**x[YFW]**R**x{20,40}[**H**RK][**D**E] reported in Reference [17] and in the expanded PDE motif [AYFW]Hx[LEYFW]Rx{20,40}[HRK][DE]x{60,90}Rx{3}[YFW] constructed in this study are labeled black and green, respectively. These amino acids, which could interact with cAMP at the PDE center, are colored according to their charges in the surface model and as individual atoms in the ribbon model. The BDPDE1^R80-A315^ 3D structure modeled against the crystal structure of a metal-dependent HD domain-containing hydrolase from *B. halodurans* (PDB ID: 3DTO) using MODELLER (ver. 9.25) [39] and cAMP docking simulations were performed using AutoDock Vina (ver. 1.1.2) [40]. Molecular graphics and analyses were performed with the UCSF Chimera package [41]. Chimera was developed by the Resource for Biocomputing, Visualization, and Informatics at the University of California, San Francisco (supported by NIGMS P41-GM103311). (**B**) The effect of site-directed mutagenesis on PDE activity. Mutations of H155G, D156G and Y237E in the wild-type (WT) BDPDE1 domain significantly reduced the enzyme activity, while L124E did not affect the activity. The reaction time was 25 min, and the standard reaction mixture contained 0.1-mM cAMP and 5 µg of the mutated protein. Different letters indicate significantly different values as compared to the control sample (*p* < 0.05).

Based on the BDPDE1 model, the PDE center is solvent-exposed and occupies a distinct cavity that could dock cAMP with a binding affinity of −4.9 kcal/mol, as predicted by molecular docking simulations (Figure 7A). Of the key amino acids in the original PDE motif [YFW]**H**x[YFW]**R**x{20,40}[**H**RK][**D**E] reported in Reference [17], only H122, R125, H155 and D156 are present in the PDE center (labeled black), while the additional amino acids R233 and Y237 (labeled green) that could also interact with cAMP are found in positions 77 and 81 downstream of D156, respectively (Figure 6B). These amino acids are not only spatially close to the substrate, but they also orientate toward cAMP at the PDE center (Figure 7A), thus implying that they could participate in key catalytic functions. The structural analysis not only provides confidence that the predicted PDE center in BDPDE1 could bind cAMP, which is the first step for catalysis and, presumably, also the subsequent hydrolysis of cAMP to 5′AMP, but also guides mutagenesis experiments to probe the role of the key amino acids in the PDE motif.

Based on our sequence and structural analyses, we identified L124, H155, D155 and Y237 as possible loss-of-function mutations to glycine (G) or glutamine (E), since these residues are spatially close to, and orientate towards, the substrate and are present in monocots, as well as in dicots and/or bacterial orthologs. The mutagenesis of the key amino acids H155 and D156, which appeared in the original PDE motif reported in Reference [17], reduced the enzymatic activity by four-fold, generating only 0.604 and 0.572-nmol AMP min^−1^ mg protein^−1^, respectively. While L124E mutagenesis did not significantly affect the catalytic efficiency, the BDPDE1^Y237E^ mutant, however, generated only 0.5-nmol AMP min^−1^ mg protein^−1^, which is approximately five-fold lower than the wild-type BDPDE1 (Figure 7B). This is consistent with our structural evaluations, which also revealed the possibility of R233 and Y237 being located downstream of the original PDE motif to interact with cAMP. Significantly, we found that these amino acids also appear downstream in bacterial orthologs, and this further justifies their inclusion into an expanded PDE motif (Figure 6B).

In the bacterial orthologous of BDPDE1, A is present in position 1 of the PDE motif, much like in monocot PDE candidates, but in position 4, E is present in place of L (Figure 6B). Thus, considering the sequence, biochemical and structural analyses in our study, we included the A and L/E amino acids, which are conserved in monocot and bacterial PDE candidates in positions 1 and 4 of the PDE motif, as well as the R and Y amino acids located downstream of the HD domain, to yield a more comprehensive and inclusive PDE motif [**A**YFW]**H**x[**L**EYFW]**R**x{20,40}[**H**RK][**D**E]x{60,90}**R**x{3}[**Y**FW] (Figure 6C). This motif can be broadly applied to discover PDEs not just in monocots but, also, in other organisms from prokaryotes and eukaryotes and identifies 25 putative PDEs in the *B. distachyon* proteome (Supplementary Table S1). Further bioinformatics and experimental characterizations of these candidates will afford a more complete understanding of the cyclic nucleotide signaling in plants and beyond.

## 3. Materials and Methods

### 3.1. Expression Vector Construct

The total RNA was isolated from leaves and stalk of *B. distachyon* using the RNeasy Plant Mini Kit (Qiagen, Hilden, Germany). The first-strand cDNA for RT-PCR was synthesized using the GoScript™ Reverse Transcription System (Promega, Madison, WI, USA) following the manufacturer’s instructions. In order to construct the expression plasmid for the GST-BDPDE1, the cDNA fragment was amplified by RT-PCR using specific primers:

-*BDPDE1* (forward)
5′-GGATCCCCAGGAATTCCCATGTGGCCAGCATCCAAAACAC-3′
-*BDPDE1* (reverse)
5′-GATGCGGCCGCTCGAGAATCAAGCCCTGCCACTCCAC-3′

The PCR reactions were performed with cDNA as the template, forward and reverse primers and CloneAmp HiFi PCR Premix (Takara Bio USA, Mountain View, CA, USA). The amplified DNA fragments were purified using an Agarose-Out DNA Purification Kit (Eurx, Gdańsk, Poland). The amplified PCR products were cloned into the pGEX-6P-2 vector (Cytiva, Uppsala, Sweden) in the EcoRI–XhoI restriction sites using an In-fusion Cloning kit (Takara Bio USA, Mountain View, CA, USA).

### 3.2. Site Directed Mutagenesis of BDPDE1

GST-BDPDE1(L124E), GST-BDPDE1(H155G), GST-BDPDE1(D156G), GST-BDPDE1(Y237E) and GST-BDPDE1(L52K) mutants were constructed by site-directed mutagenesis using the QuikChange II XL Site-Directed Mutagenesis Kit (Agilent, Cedar Creek, TX, USA). Specific primers used in the reaction are in the Supplementary Materials.

### 3.3. Expression and Purification of the Recombinant Protein

The resulting plasmids were introduced into *E. coli* BL21(DE3) pLysS-competent cells (Promega, Madison, WI, USA) in order to produce the fusion proteins with a glutathione-S-transferase (GST) affinity tag. The transformants were grown in LB medium (500 mL) containing ampicillin (100 μg/mL) and 2% glucose at 37 °C. Fusion protein expression was induced by adding isopropyl-ß-D-thiogalactopyranoside (IPTG) to a final concentration of 0.5 mM at OD_600_ = 0.6 and incubating the culture at 20 °C for 3.5 h. The bacteria were harvested by centrifugation, and the pellet was suspended in lysis buffer (50-mM Tris-HCl, pH 8.0, 150-mM NaCl, 5-mM EDTA, 5-mM EGTA, 1 % (*v/v*) Triton X-100, 1-mM PMSF and 0.2-mg/mL lysozyme) and disrupted by sonication. The cell extract was centrifuged at 18,000× *g* for 35 min, and the supernatant was loaded onto glutathione-Sepharose 4B beads (Cytiva, Uppsala, Sweden). Afterward, the column was washed multiple times with a buffer containing 50-mM Tris-HCl (pH 8.0) and 150-mM NaCl, and the GST fusion protein was eluted with 10-mM glutathione in 50-mM Tris-HCl (pH 9.0). The recombinant calmodulins (CaM1, 3, 7 and CML9) were purified according to Reference [32]. The homogeneity and purity of the eluted protein fraction was analyzed by SDS–PAGE electrophoresis (10% gel) with the Coomassie Blue gel staining.

### 3.4. Structural Analysis of the PDE Center and CaM-Binding Site in BDPDE1

The BDPDE1^R80-A315^ 3D structure was modeled against the crystal structure of a metal-dependent HD domain-containing hydrolase from *B. halodurans* (PDB ID: 3DTO) using MODELLER (ver. 9.25) [39], and cAMP docking simulations were performed using AutoDock Vina (ver. 1.1.2) [40]. In the docking simulations, all bonds in the cAMP were allowed to move freely, but BDPDE1^R80-A315^ was set as rigid. Docking simulations consider both spatial and charge at the vicinity of the PDE center based on predetermined grids that cover the entire catalytic center. Molecular graphics and analyses were performed with the UCSF Chimera package [41]. Chimera was developed by the Resource for Biocomputing, Visualization, and Informatics at the University of California, San Francisco (supported by NIGMS P41-GM103311).

### 3.5. PDE Biochemical Assay and LC-MS/MS Analysis

PDE in vitro activity was determined by using LC-MS/MS to determine the rate of AMP or GMP formation. The reaction time was 25 min, and the standard reaction mixture contained: 3-mM Tris-HCl (pH 8.0), 0.1-mM cAMP or cGMP, 0.1% (*v/v*) 2-mercaptoethanol, 5 µg of GST-BDPDE1 and 0.5-mM MgCl_2_ and MnCl_2_ in a final volume of 100 μL. To investigate if calmodulin regulates the activity of PDE, four different CaM isoforms were added to the reaction in the concentration of 2-µM, together with 12.5-μM CaCl_2_, and the GST-BDPDE1 protein concentration was 0.625-µM. The concentration of free Ca^2+^ ions was calculated using the Maxchelator program available online at: maxchelator.stanford.edu [42]. The samples were incubated at 37 °C for 25 min. The enzyme reaction was terminated by incubation at 100 °C for 10 min, and the samples were centrifuged at 13,200× *g* for 10 min.

LC-MS/MS experiments were performed using the Nexera UHPLC and LCMS-8045 integrated systems (Shimadzu Corporation, Kyoto, Japan). The ionization source parameters were optimized in positive ESI mode using pure AMP and GMP dissolved in HPLC grade water (Sigma, St. Louis, MO, USA). The samples were separated using a XSelect CSH Phenyl-Hexyl column (100 × 2.1 mm, 3.5 µm, Waters, Dublin, Ireland). A gradient of solvent A (0.05 % (*v/v*) formic acid with 5-mM ammonium formate) and solvent B (100 % (*v/v*) acetonitrile) was applied over 3 min: B: 0–5%, followed by washing and conditioning of the column with a flow rate of 0.4 mL/min. The interface voltage was set at 4.0 kV for positive (ES+) electrospray. Data acquisition and analysis were made with the LabSolutions workstation for LCMS-8045.

### 3.6. Fluorescence Studies

The fluorescence spectra of plant phosphodiesterase (BDPDE1) in the absence and presence of CaM1 and CML9 in 10-mM glutathione and 50-mM Tris-HCl (pH 9.0) were performed on a JASCO FP-8300 spectrofluorometer with 10-mm quartz cells (Hellma Analytics, Müllheim, Germany). The measurements were recorded in the range of 300–600 nm after excitation at λ = 280 nm at 37 °C. The samples were prepared in 2-mL Eppendorf tubes and contained BDPDE1 at a concentration of 0.5-μM alone or with CaM1 and CML9 at the following concentrations: 0.25, 0.5, 1.0, 1.5, 2.0 and 2.5 μM, and 10-mM glutathione in 50-mM Tris-HCl (pH 9.0) was added to each tube, up to 2 mL. Then, the spectrum was recorded, and the emission spectra were measured three times. The fluorescence data were fitted by applying nonlinear least-squares regression using OriginPro software Version 2016 (OriginLab Corporation, Northampton, MA, USA).

### 3.7. Statistical Analyses

All experiments were performed in at least triplicate. Values are expressed as the mean ± SE. Differences between the groups were calculated by one-way ANOVA, followed by a Tukey–Kramer multiple comparison test using SigmaPlot 11.0 software. In all cases, the confidence coefficient was set at *p* < 0.05.

## 4. Conclusions

Degradation of cNMPs by PDEs is an integral component of cyclic nucleotide-dependent signaling in organisms across the tree of life; yet, plant PDEs have remained largely elusive. Given the recent discovery of PDEs in dicots [16,17], we set out to identify novel PDEs in monocots. Our results showed that a *B. distachyon* ortholog of the *A. thaliana* PDE ATCN-PDE1, BDPDE1, can hydrolyze cNMPs to 5′NMPs with a preference for cAMP over cGMP in vitro, and importantly, the PDE activity was significantly enhanced by the CaM/Ca^2+^ complex. Through bioinformatics-guided mutagenesis studies, we also ascertained the key residues involved in both PDE catalytic activity and in the interaction of CaM. Our results imply that plant PDE domains may be embedded within complex multidomain proteins where they are likely to modulate intra- and intermolecular domains, thereby acting as tuners of downstream signals. Finally, based on our biochemical, mutagenesis and structural analyses, we constructed a comprehensive amino acid consensus sequence that is a diagnostic for annotated and/or experimentally validated PDEs across kingdoms, thus affording broad applications of this search motif for the identification of PDE active sites in eukaryotes and prokaryotes.

## Data Availability

The data is contained within the article and the Supplementary Materials.

## Supplementary Materials

The following are available online at https://www.mdpi.com/article/10.3390/ijms22179654/s1.
