# Supplementary material for: In Search of Monocot Phosphodiesterases: Identification of a Calmodulin Stimulated Phosphodiesterase from Brachypodium distachyon
Source: Int J Mol Sci. 2021 Sep 6;22(17):9654. doi: 10.3390/ijms22179654 (PMC8431786; doi:10.3390/ijms22179654)
Supplement: Supplementary file 1 [file ijms-22-09654-s001.zip › ijms-1358632-supplementary.pdf]

Supplementary Information

In Search of Monocot Phosphodiesterases: *Identification* of a Calmodulin Stimulated Phosphodiesterase from *Brachypodium distachyon*

Mateusz Kwiatkowski[1]*, Aloysius Wong[2, 3], Anna Kozakiewicz-Piekarz[4], Christoph Gehring[5], Krzysztof Jaworski[1]

[1] Chair of Plant Physiology and Biotechnology, Nicolaus Copernicus University, Lwowska St. 1, 87-100 Torun, Poland

[2] Department of Biology, College of Science and Technology, Wenzhou-Kean University, 88 Daxue Road, Ouhai, Wenzhou, Zhejiang Province 325060, China

[3] Zhejiang Bioinformatics International Science and Technology Cooperation Center of Wenzhou-Kean University

[4] Department of Biomedical and Polymer Chemistry, Faculty of Chemistry, Nicolaus Copernicus University, Gagarina St. 7, 87-100 Torun, Poland

[5] Department of Chemistry, Biology and Biotechnology, University of Perugia, Borgo XX giugno, 74, 06121 Perugia, Italy

*Corresponding author: Tel: +48-56-611-44-56; Fax: +48-56-61-14-772; E-mail: m.kwiatkowski@doktorant.umk.pl

Supplementary Information contains 2 figures, 1 table and supplementary methods

Supplementary Figure S1

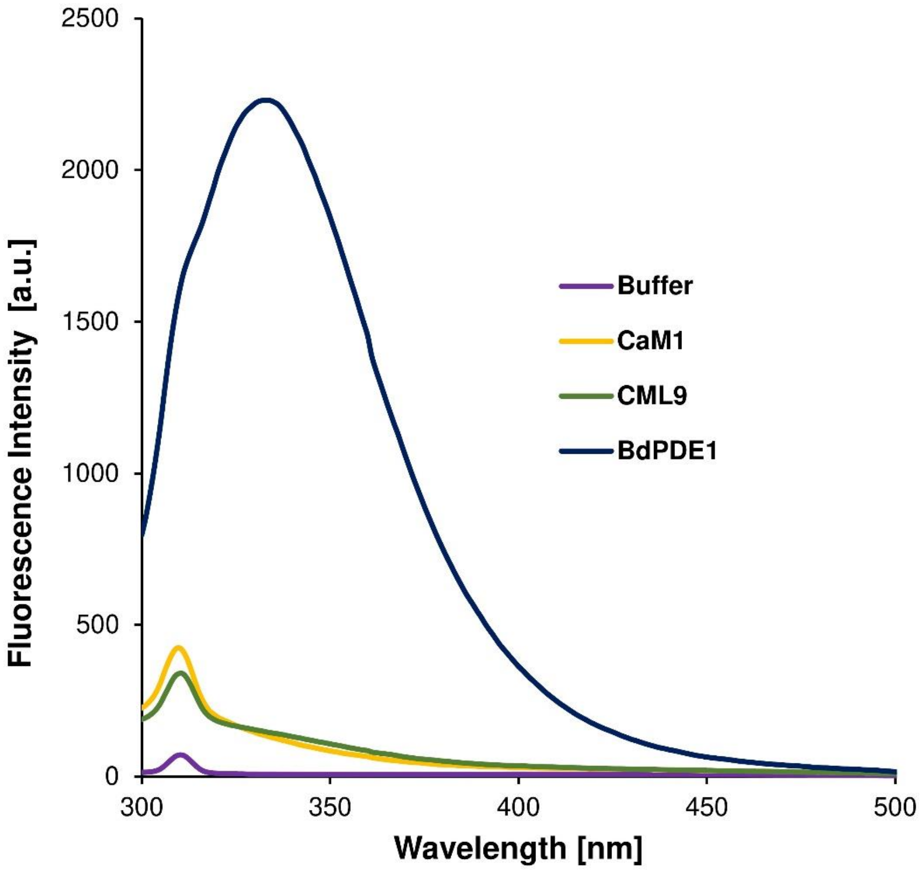

Emission spectra of 0.5 μM BDPDE1, 10 mM glutathione in 50 mM Tris-HCl (pH 9.0), 0.5 μM CaM1 and 0.5 μM CML9.

Supplementary Figure S2

A

```
AtCaM1      MADQLTDEQISEFKEAFSLFDKDGDGCITTKELGTVMRSLGQNPTEAELQDMINEVDADG     60
BdCaM1      MADQLTDEQIAEFKEAFSLFDKDGDGCITTKELGTVMRSLGQNPTEAELQDMINEVDADG     60
            **********:*************************************************

AtCaM1      NGTIDFPEFLNLMAKKMKDTDSEEELKEAFRVFDKDQNGFISAAELRHVMTNLGEKLTDE     120
BdCaM1      NGTIDFPEFLNLMARKMKDTDSEEELKEAFRVFDKDQNGFISAAELRHVMTNLGEKLTDE     120
            **************:*********************************************

AtCaM1      EVEEMIREADVDGDGQINYEEFVKIMMAK    149
BdCaM1      EVDEMIREADVDGDGQINYEEFVKVMMAK    149
            **:*********************:****
```

B

```
AtCaM3      MADQLTDDQISEFKEAFSLFDKDGDGCITTKELGTVMRSLGQNPTEAELQDMINEVDADG     60
BdCaM3      MADQLTDDQIAEFKEAFSLFDKDGDGCITTKELGTVMRSLGQNPTEAELQDMINEVDADG     60
            **********:*************************************************

AtCaM3      NGTIDFPEFLNLMARKMKDTDSEEELKEAFRVFDKDQNGFISAAELRHVMTNLGEKLTDE     120
BdCaM3      NGTIDFPEFLNLMARKMKDTDSEEELKEAFRVFDKDQNGFISAAELRHVMTNLGEKLTDE     120
            ************************************************************

AtCaM3      EVDEMIKEADVDGDGQINYEEFVKVMMAK    149
BdCaM3      EVDEMVREADVDGDGQINYDEFVKVMMAK    149
            *****::************:*********
```

C

```
AtCaM7      MADQLTDDQISEFKEAFSLFDKDGDGCITTKELGTVMRSLGQNPTEAELQDMINEVDADG     60
BdCaM7      MADQLTDDQIAEFKEAFSLFDKDGDGCITTKELGTVMRSLGQNPTEAELQDMINEVDADG     60
            **********:*************************************************

AtCaM7      NGTIDFPEFLNLMARKMKDTDSEEELKEAFRVFDKDQNGFISAAELRHVMTNLGEKLTDE     120
BdCaM7      NGTIDFPEFLNLMARKMKDTDSEEELKEAFRVFDKDQNGFISAAELRHVMTNLGEKLTDE     120
            ************************************************************

AtCaM7      EVDEMIREADVDGDGQINYEEFVKVMMAK    149
BdCaM7      EVDEMIREADVDGDGQINYEEFVKVMMAK    149
            *****************************
```

D

```
AtCaM1      MADQLTDEQISEFKEAFSLFDKDGDGCITTKELGTVMRSLGQNPTEAELQDMINEVDADG     60
AtCML9      MADAFTDEQIQEFYEAFCLIDKDSDGFITKEKLTKVMKSMGKNPKAEQLQQMMSDVDIFG     60
            *** :*****.** ***.*:***.** **.::* .**:*:*:**.  :**:*:.:**  *

AtCaM1      NGTIDFPEFLNLMAKKMKDTDSEEELKEAFRVFDKDQNGFISAAELRHVMTNLGEKLTDE     120
AtCML9      NGGITFDDFLYIMAQNTSQESASDELIEVFRVFDRDGDGLISQLELGEGMKDMGMKITAE     120
            ** * * :** :**:: .: .:.:** *.*****:* :*:**  ** . *.::* *:* *

AtCaM1      EVEEMIREADVDGDGQINYEEFVKIMMAK--  149
AtCML9      EAEHMVREADLDGDGFLSFHEFSKMMIAASY  151
            *.*.*:****:**** :.:.** *:*:*
```

Sequence comparison of calmodulins derived from *A. thaliana* and *B. distachyon*. The CaM 1, CaM3 and CaM7 alignments are shown in figure 1A, B and C, respectively. D is the alignment of CaM1 and the calmodulin-like protein 9 (CML9) derived from *A. thaliana*.

Supplementary Methods

Specific primers used in site-directed mutagenesis

- *BDPDE1*^{L124E} (forward)

5′- GCGGACGCGGCGCGCGTGGGC -3′

- *BDPDE1* ^{L124E} (reverse)

5′- GCCCACGCGCGCCGCGTCCGC -3′

- *BDPDE1*^{H155G} (forward)

5′- TAATCCCCGATGTCGCCCAGCAGAGCAGCCAG -3′

- *BDPDE1* ^{H155G} (reverse)

5′- CTGGCTGCTCTGCTGGGCGACATCGGGGATTA -3′

- *BDPDE1* ^{D156G} (forward)

5′- CCTTGGTATACTTGTAACCCCCGATGTCGTGCAGC-3′

- *BDPDE1* ^{D156G} (reverse)

5′- GCTGCACGACATCGGGGGTTACAAGTATACCAAGG-3′

- *BDPDE1* ^{Y237E} (forward)

5′- TTCTTGCTCCCTCCTTCTGTAAAACATCTTGCAACACCAATTGC-3′

- *BDPDE1* ^{Y237E} (reverse)

5′- GCAATTGGTGTTGCAAGATGTTTTACAGAAGGAGGGAGCAAGAA-3′

- *BDPDE1* ^{L52K} (forward)

5′- CGCTTCGTCGGACTCGAAGTTTACGATTCTCGATCTCCCTA-3′

- *BDPDE1* ^{L52K} (reverse)

5′- TAGGGAGATCGAGAATCGTAAACTTCGAGTCCGACGAAGCG-3′

Supplementary Table S1

List of *B. distachyon* candidate PDEs

Search pattern [AYFW] H x [LEYFW] R x {20,40} [HRK] [DE] x {60,90} R x {3} [YFW]
Dataset searched *Brachypodium distachyon* proteome ID UP000008810

Gene ID	Region	Hit pattern	Description
A0A0Q3H5X5	353-481	FHMWRMVSVNQSYGYGQQLRSIPSQPHCHLRF VDITGFYGQKDQLELALHILRNAAMLEAMKID PKPSIAAEYGQMQGPFFLDGYQVARDYVLRED KCNVLYIKHVPRKTIEAKFLSGSLWSKLRTLSY	F-box domain containing protein
I1II25	295-417	AHPERIQKLVKNYSQFVSFPIYTWQEKGFTKEV EVDEDPAAAKTEGDGDTEKQAKKKTKTVVEK YWDWELTNETQPIWLRNPREVSTEEYNEFYKK TFNEYLDPLASSHFTTEGEVEFRSILY	HATPase cdomain containing protein
I1HVY3	127-237	FHKERYNKDVKEKERLKTKSAIVLRGGENKEV RFKGKTEIKDLKVKADAITRLMERGYRVKCMA MPAGNEEEDLGGPLSRLLGLIQDVCIVESGPHL DSKHAYVIVRHVKF	IF3_N domain containing protein
I1ITQ2	526-630	AHRYRHLITNDQLFRPVLDPQGWDHDIGFDGI NFESSHELKRNISSSIAGQMRKDKEDMYIQSEC SVSYTDQRGYSLMGGMDMQTASKDLVCTVHG DAKYRNFPW	AIG1-type G domain containing protein
I1GWH8	158-285	AHQFRWLLSQVNYPKLGDLCWLVIPCALTALD HWSPDVKEQGVVSFMHIAKNVKVTELSLYED AILDACCNYIPADDDLWYHIVEVSVLLLTCTQR SNPRSPWYDRMLSAMLGHLERQPLNQKRRIW	Uncharacterized protein
A0A0Q3GZJ1	142-262	FHQLRSGTSSSVSSQDGDSNERSTDDETEKEEV NADNEHDEEGVKVVAWSADDQKNILKIGCLEI ERNQRLEMLIARHRVRKDVDRNLIDFGSGDSIP TVEELSKFNVQIPTVFAPRRNPF	Uncharacterized protein
I1H012	2-108	AHEWRRAAASASAAALDAAEDALFVDIAHEA PLSCQRQSQSIVGGTLYCILLAGYAGVAIAAPW IFVLIPEMTLPLLCSCNVLLLIVTGIFQQYWVHQ VTKVRLQGY	Uncharacterized protein
A0A2K2DUJ7	168-289	AHRLRPPRRVTALVPLDALDAEKLYRRRFARD VEFFPTDIGAVLGNTLSLGTFLAVVVGAEDASK KFEWRGVEQFLASPPASWAVASLWDCGGVFR LEMRGASRARRALAAASRALDRAAKW	N-acetyltransferase domain protein
A0A0Q3GP66	245-353	AHEWRYVVENQCMLWEQRDGEGVVPEPGRV GRHERPVQVAGAGQLLPGPPKLPGGVQGQLG AAAGHARRLPRRRREPMGELRRRRFLQKKRW RRRGGGHRQGQRRAGLW	PLCXc domain containing protein
A0A0Q3GWX6	103-215	AHSLRGFEAIDTAKAALELACPGVVSCADVLA FAARDASYILSVGNYNEPIEFAMPGGRLDGRRS MAEDTLHGSLPSASANVTELVDAFQTKGLGVE DLVVLSGAHSARCSFF	Peroxidase

A0A0Q3FAV4	121-233	AHRWRDAAAAAPSSCPASSTAAIGGGILPPTTA SLARRETPPPPPPRPPTGASPPAPSTAAASFPSA WRRPCDGGSGEARVLRRQLRGQQAWSAPWTA TAPLPAVHSSRVYKY	Uncharacterized protein
I1IA86	215-329	AHVYRSRDFKTWKRAKHPLHSALTGMWECPD FFPVHEAGVQNGLETSQFAAKYVLKNSLDLTR YDYYTVGTYNNKTDRYVPDDANGDYRRLQY DYGNFYASKTFYDPAKQRRVLF	Uncharacterized protein
A0A0Q3FTD3	265-383	WHEERCVRASNITIDARMLEVSGLPCRPPPRDH SNDEAAAQAQQQLEFHNLSMCQPALCPNGED DVVYLLAREKYLHPKAWCLAVDMKNQGTLK HVAYIGNQGHPFCHRIFCLSRISKY	DUF1618 domain containing protein
A0A0Q3IC40	279-394	AHTWRAVSRARRLPPTEETTYTVLVGCRDRVK GVPRDYAGNAVVRATARSTAGEILGHGLGWT ARLLNRAVASSGDEAAVRGFLGRWAREPRFA YLAGFWNPAMVVTGNSPRFDAF	Uncharacterized protein
I1J2V7	33-148	WHAERAASSAAQPASGTRSQYNVELESVNRFL DGILREKPARFTAENLREFTGGYAERVGSGGFG VVYRGRFPNGVAVAVKVLNGTLDRRAEEQFM AEVGTAGRTYHINLVRLYGF	Protein kinase domain protein
I1HF09	665-781	FHLRRGPLLGSIVGHEDERSVLRNLFLNASFDL SLRMLAPRCIMHREGGTFEELPAYDLAMQSYS AVVLDHGTDIFIWLGAELATKEGQSAAALAAC RTLAEELSELRFPAPRILSF	Protein transport SEC23
I1GRD2	66-193	AHALRRPIEVLLYTRPVHGCGEEDLGLDALAV VSGSGDRDRRRRLASIPAMPHRCGPRPCCCLLA SCDGLLLLGHGRGNEFQPYASYLVCNPATRQW TQLPRVTAEFAAAGKRPGHGPARLEFRESGF	F-box domain containing protein
I1HUJ6	662-778	AHTLRFEAYELPKPSLPTATSQTSISLPTTDLVP VPEQSYYKDDHQMSRPQPSGNALSGEFGTKLR LDGVQKKWGRESYSSSSTPSSSTSSQQAANGST NSDGGGLVVSSQARESSY	AP4 complex subunit epsilon
**A0A0Q3LY42**	121-237	AHALRVRDLALSLAAEQGLSSPDRLLIVELAAL LHDIGDYKYTKDNMEDMSIVERFLQDVGLEKG QREEIVSIITGMGFKNEVSGKAAEPTLEFAVVQ DADRLDAIGAIGVARCFTY	BdPDE1, HD domain containing protein
A0A0Q3GTT5	173-293	AHLFRSWATFLNRSIILTPEGDRTDKRGISAFNT WKDIIIPGNVDDSMVKPDARAVQPIPLTKRKYL ANFLGRAQGKAGRLKLVELAKQYPDKLESPEL KLSGPDKLGRIDYFKHLRNAKF	Exostosin domain containing protein
I1GZC4	244-351	FHPERGSSTADLADDSRFIEFYNLVFMQYNKK DDGSLEPLKQKNIDTGMGLERMARILQNVPNN YETDLIFPIIKKAASMALVSYSTADDTMKTNLK IIGDHMRAVVY	Probable alanine tRNA ligase
I1HR05	55-164	AHYRRRCRIRAPCCNEIFDCRHCHNEAKNSIGI DTMRRHEIPRHEVQQQVICSLCGTEQEVRQICI NCGVCMGKYFCEVCKLFDDDVSKQQYHCHGC GICRIGGRENFF	Uncharacterized protein

A0A2K2CIP6	381-510	WHVWRSQIHNNNNGGFDFADPEWLDAAFLFY DEEARLVRVRVRDCLDMASLGYAYQEVPLPW LDARPTKPASPGTPARALDALPATLDKTVRVA VARPRASRSAKEKEEQEEVVVVEGIQVSDCSRF VKF	Uncharacterized protein
A0A2K2D3H0	58-190	FHHFRALISFDSISSIKSQLENGSAKPNWTQSSPP RPRDHHDLRHSSVLRRRRRRRVTARPHSRAPLP GVPDYPRRKFALGCIRGTMPLFMKKPFSLLEPP KDLDPKEKVFQIRFTKEIFLDYQEYVKRLNLY	Uncharacterized protein
I1IFT6	227-338	FHALRNTRGLPWPKEHEKKSDADLLDWLQAM FGFQTDSVSNQREHLILLLANMHIRQISKPDQQ SKLDDGALDKVMKKLFKNYKRWCKYLGRKSS LRLPTIQQEVQQRKLLY	1,3-beta-glucansynthase
